# (2*S*)-Ethyl 2-[(*S*_s_)-benzyl­sulfinyl­amino]-3,3-dimethylbutanoate

**DOI:** 10.1107/S1600536808028717

**Published:** 2008-09-13

**Authors:** Wei Zheng, Xun Sun, Jie Sun, Bang-Guo Wei

**Affiliations:** a Department of Chemistry of Natural Drugs, School of Pharmacy, Fudan University, Shanghai 200032, People’s Republic of China; b Shanghai Institute of Organic Chemistry, Shanghai 200032, People’s Republic of China; c Department of Chemistry, Fudan University, Shanghai 200032, People’s Republic of China

## Abstract

The title compound, C_15_H_23_NO_3_S, is an unexpected 1,3-migration product in the addition of benzyl­zinc bromide to *N*-*tert*-butane­sulfinyl imino­acetate. In the crystal structure, mol­ecules are linked by N—H⋯O hydrogen bonds and weak C—H⋯O hydrogen bonds.

## Related literature

For general background, see: Ellman *et al.* (2002 ▶); Lin *et al.* (2008 ▶); Daniel & Stockman (2006 ▶). For the synthesis of the titled compound, see: Sun *et al.* (2008 ▶).
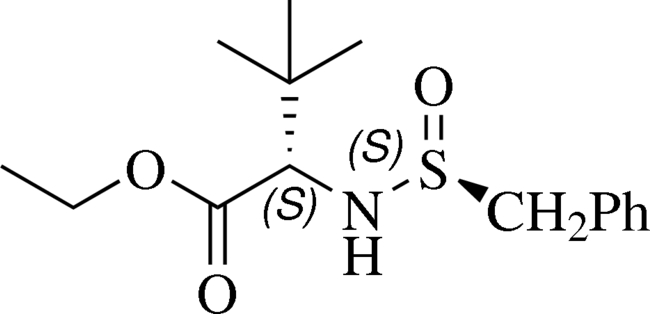


## Experimental

### 

#### Crystal data

C_15_H_23_NO_3_S
*M* *_r_* = 297.40
Monoclinic, 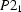

*a* = 11.166 (2) Å
*b* = 7.1917 (14) Å
*c* = 11.460 (2) Å
β = 115.473 (3)°
*V* = 830.8 (3) Å^3^
*Z* = 2
Mo *K*α radiation
μ = 0.20 mm^−1^
*T* = 293 (2) K
0.49 × 0.41 × 0.17 mm

#### Data collection

Bruker SMART APEX CCD area-detector diffractometer
Absorption correction: multi-scan (*SADABS*; Bruker, 2001 ▶) *T*_min_ = 0.908, *T*_max_ = 0.967
4782 measured reflections
3221 independent reflections
2661 reflections with *I* > 2σ(*I*)
*R*_int_ = 0.120

#### Refinement

*R*[*F* ^2^ > 2σ(*F* ^2^)] = 0.054
*wR*(*F* ^2^) = 0.134
*S* = 0.97
3221 reflections
189 parameters
2 restraints
H atoms treated by a mixture of independent and constrained refinement
Δρ_max_ = 0.43 e Å^−3^
Δρ_min_ = −0.23 e Å^−3^
Absolute structure: Flack (1983 ▶), 1295 Friedel pairs
Flack parameter: −0.09 (11)

### 

Data collection: *SMART* (Bruker, 2001 ▶); cell refinement: *SAINT* (Bruker, 2001 ▶); data reduction: *SAINT*; program(s) used to solve structure: *SHELXS97* (Sheldrick, 2008 ▶); program(s) used to refine structure: *SHELXL97* (Sheldrick, 2008 ▶); molecular graphics: *SHELXTL* (Sheldrick, 2008 ▶); software used to prepare material for publication: *SHELXTL*.

## Supplementary Material

Crystal structure: contains datablocks I, global. DOI: 10.1107/S1600536808028717/zl2133sup1.cif

Structure factors: contains datablocks I. DOI: 10.1107/S1600536808028717/zl2133Isup2.hkl

Additional supplementary materials:  crystallographic information; 3D view; checkCIF report

## Figures and Tables

**Table 1:** Hydrogen-bond geometry (Å, °)

*D*—H⋯*A*	*D*—H	H⋯*A*	*D*⋯*A*	*D*—H⋯*A*
C7—H7*B*⋯O1	0.96	2.61	3.234 (5)	123
C9—H9*B*⋯O3^i^	0.97	2.48	3.296 (5)	142
N1—H1*A*⋯O3^i^	0.859 (17)	2.13 (2)	2.932 (3)	156 (3)

Symmetry code: (i) 
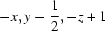
.

**Fig. 1.**
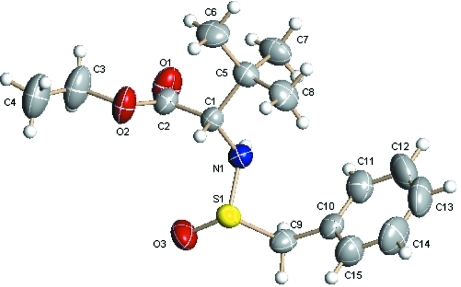
Plot of C15H23NO3S with 50% probability levels. H atoms are drawn as spheres of arbitrary radius.

**Fig. 2.**
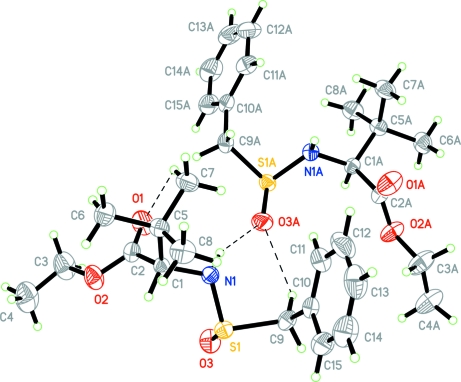
Hydrogen bonding in the title compound. Symmetry code: (i) -x, y-1/2, -z+1.

**Fig. 3.**
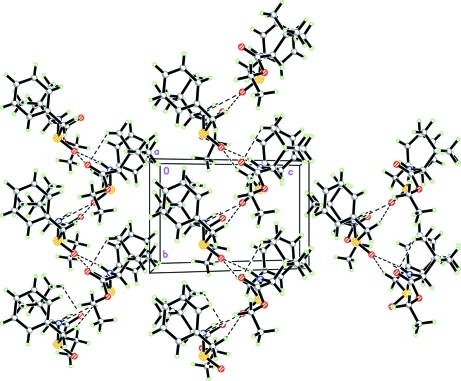
Molecular packing plot showing the one-dimensional polymeric chains of the title compound. Hydrogen bond interactions are shown as dashed lines.

**Fig. 4.**
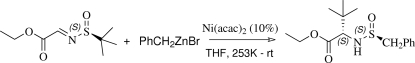
Reaction sequence.

## supplementary crystallographic information

### Comment

*N*-*tert*-Butanesulfinylamide has received considerable attention in the auxiliary-aided asymmetric synthesis of a broad range of chiral amines (Ellman *et al.*, 2002; Stockman *et al.*, 2006; Lin *et al.*, 2008). In our research on the asymmetric addition of organozinc reagents to chiral *N*-*tert*-butanesulfinyl iminoacetates, an unexpected rearrangement product was obtained instead of the desired nucleophilic addition product. The structure of the compound obtained by 1,3-migration of the *tert*-butyl group was determined to be (2*S*)-ethyl 3,3-dimethyl-2-((*S*_s_)-benzylsulfinylamino)butanoate. The reaction sequence (Sun *et al.*, 2008) is briefly shown in Fig. 4. The absolute configuration at the sulfur atom (as determined by the Flack parameter) is *S* as in the starting material. The new chiral center at C1 also exhibits an *S*-configuration. We believe this unusual rearrangement reaction could be developed to be a novel and convenient approach to prepare *tert*-leucine.

The crystal packing in the title compound is stabilized by an intramolecular hydrogen interaction (C7—H7B···O1) and by two intermolecular hydrogen bonds (N1—H1A···O3^i^ and C9—H9B···O3^i^, symmetry operator: (i) -x, y-1/2, -z+1) which lead to the formation of an one-dimensional hydrogen bonded chain along the b axis as shown in Fig. 3.

### Experimental

To a solution of ethyl *N*-(*tert*-butanesulfinyl)iminoacetate (1 mmol) and Ni(acac)_2_ (10 mol%) in anhydrous THF (10 ml) was added freshly prepared benzylzinc bromide (2.5 ml, 1 *M* in THF) at 195 K under an argon atmosphere. Then the mixture was allowed to warm to room temperature. After stirring for another 6 h, the reaction was quenched with saturated aqueous NH_4_Cl (4 ml). The mixture was extracted with EtOAc (10 ml) twice. The combined organic phases were washed with brine and dried with anhydrous Na_2_SO_4_. After concentrating under reduced pressure, the residue was purified by silica gel chromatography to give the title compound (yield: 47%). Suitable crystals were obtained by recrystallization from acetone (m.p. 421–423 K). [α]_D_^25^ 132.2 (c = 0.60, CHCl_3_). ^1^H NMR (δ, CDCl_3_) 7.31-7.42 (*m*, 5H), 4.33 (*d*, *J* = 9.0, 1H), 4.12-4.20 (*m*, 2H), 4.03 (*s*, 2H), 3.48 (*d*, *J* = 9.0, 1H), 1.24 (*t*, *J* = 7.0, 3H), 0.83 (*s*, 9H). HRMS for (C_15_H_23_NO_3_S) found 289.1469, Calcd 289.1477.

### Refinement

Hydrogen atoms bonded to carbon were generated geometrically (C—H = 0.93, 0.98, 0.97 or 0.96 Å for phenyl, tertiary, methylene or methyl H atoms respectively) and refined in the riding model approximation. The hydrogen atom bound to the N atom was located from a difference density Fourier map, was refined isotropically and the N—H distance was restrained to 0.86 (2) Å. The displacement parameters of methyl H atoms were set to 1.5 times *U*_eq_ of the equivalent isotropic displacement parameters of their parent atoms, while those of other H atoms bound to C were set to 1.2 times *U*_eq_.

### Crystal data

**Table:**

C_15_H_23_NO_3_S	*F*(000) = 320
*M**_r_* = 297.40	*D*_x_ = 1.189 Mg m^−^^3^
Monoclinic, *P*2_1_	Mo *K*α radiation, λ = 0.71073 Å
Hall symbol: P 2yb	Cell parameters from 1845 reflections
*a* = 11.166 (2) Å	θ = 3.4–23.9°
*b* = 7.1917 (14) Å	µ = 0.20 mm^−^^1^
*c* = 11.460 (2) Å	*T* = 293 K
β = 115.473 (3)°	Prismatic, colorless
*V* = 830.8 (3) Å^3^	0.49 × 0.41 × 0.17 mm
*Z* = 2	

### Data collection

**Table:**

Bruker SMART APEX CCD area-detector diffractometer	3221 independent reflections
Radiation source: fine-focus sealed tube	2661 reflections with *I* > 2σ(*I*)
graphite	*R*_int_ = 0.120
φ and ω scans	θ_max_ = 27.0°, θ_min_ = 2.0°
Absorption correction: multi-scan (SADABS; Bruker, 2001)	*h* = −11→14
*T*_min_ = 0.908, *T*_max_ = 0.967	*k* = −8→9
4782 measured reflections	*l* = −14→13

### Refinement

**Table:**

Refinement on *F*^2^	Secondary atom site location: difference Fourier map
Least-squares matrix: full	Hydrogen site location: inferred from neighbouring sites
*R*[*F*^2^ > 2σ(*F*^2^)] = 0.055	H atoms treated by a mixture of independent and constrained refinement
*wR*(*F*^2^) = 0.134	*w* = 1/[σ^2^(*F*_o_^2^) + (0.0662*P*)^2^] where *P* = (*F*_o_^2^ + 2*F*_c_^2^)/3
*S* = 0.97	(Δ/σ)_max_ = 0.001
3221 reflections	Δρ_max_ = 0.43 e Å^−^^3^
189 parameters	Δρ_min_ = −0.23 e Å^−^^3^
2 restraints	Absolute structure: Flack (1983), 1295 Friedel pairs
Primary atom site location: structure-invariant direct methods	Flack parameter: −0.09 (11)

### Special details

**Table:**

Geometry. All e.s.d.'s (except the e.s.d. in the dihedral angle between two l.s. planes) are estimated using the full covariance matrix. The cell e.s.d.'s are taken into account individually in the estimation of e.s.d.'s in distances, angles and torsion angles; correlations between e.s.d.'s in cell parameters are only used when they are defined by crystal symmetry. An approximate (isotropic) treatment of cell e.s.d.'s is used for estimating e.s.d.'s involving l.s. planes.
Refinement. Refinement of *F*^2^ against ALL reflections. The weighted *R*-factor *wR* and goodness of fit *S* are based on *F*^2^, conventional *R*-factors *R* are based on *F*, with *F* set to zero for negative *F*^2^. The threshold expression of *F*^2^ > σ(*F*^2^) is used only for calculating *R*-factors(gt) *etc*. and is not relevant to the choice of reflections for refinement. *R*-factors based on *F*^2^ are statistically about twice as large as those based on *F*, and *R*- factors based on ALL data will be even larger.

### Fractional atomic coordinates and isotropic or equivalent isotropic displacement parameters (Å^2^)

**Table:**

	*x*	*y*	*z*	*U*_iso_*/*U*_eq_	
S1	0.04167 (7)	0.26178 (12)	0.67424 (6)	0.0442 (2)	
O1	0.3061 (3)	0.0339 (4)	0.5596 (3)	0.0741 (8)	
O2	0.4124 (3)	0.2630 (5)	0.6946 (2)	0.0696 (7)	
O3	0.0313 (3)	0.3869 (4)	0.5670 (2)	0.0665 (7)	
N1	0.1252 (2)	0.0717 (4)	0.6804 (2)	0.0420 (6)	
C1	0.2690 (3)	0.0814 (4)	0.7500 (3)	0.0397 (6)	
H1	0.2901	0.1873	0.8094	0.048*	
C2	0.3305 (3)	0.1206 (5)	0.6568 (3)	0.0501 (8)	
C3	0.4801 (5)	0.3093 (8)	0.6131 (5)	0.0923 (17)	
H3A	0.5348	0.2058	0.6108	0.111*	
H3B	0.4155	0.3353	0.5255	0.111*	
C4	0.5613 (6)	0.4705 (9)	0.6677 (5)	0.108 (2)	
H4A	0.5056	0.5775	0.6547	0.162*	
H4B	0.6189	0.4900	0.6263	0.162*	
H4C	0.6136	0.4516	0.7586	0.162*	
C5	0.3281 (3)	−0.0954 (5)	0.8332 (3)	0.0478 (7)	
C6	0.4791 (4)	−0.0865 (6)	0.8887 (4)	0.0681 (10)	
H6A	0.5167	−0.1912	0.9445	0.102*	
H6B	0.5099	0.0264	0.9371	0.102*	
H6C	0.5057	−0.0892	0.8194	0.102*	
C7	0.2792 (4)	−0.2720 (5)	0.7539 (4)	0.0649 (10)	
H7A	0.3185	−0.3781	0.8076	0.097*	
H7B	0.3039	−0.2702	0.6833	0.097*	
H7C	0.1844	−0.2791	0.7206	0.097*	
C8	0.2833 (4)	−0.0948 (6)	0.9414 (3)	0.0653 (10)	
H8A	0.1882	−0.0909	0.9048	0.098*	
H8B	0.3191	0.0124	0.9952	0.098*	
H8C	0.3145	−0.2055	0.9924	0.098*	
C9	−0.1205 (3)	0.1504 (5)	0.6172 (3)	0.0513 (8)	
H9A	−0.1880	0.2455	0.5975	0.062*	
H9B	−0.1384	0.0834	0.5380	0.062*	
C10	−0.1293 (3)	0.0198 (5)	0.7130 (3)	0.0494 (8)	
C11	−0.1140 (4)	−0.1679 (6)	0.7036 (4)	0.0664 (10)	
H11	−0.1014	−0.2145	0.6340	0.080*	
C12	−0.1169 (5)	−0.2890 (6)	0.7960 (5)	0.0895 (15)	
H12	−0.1062	−0.4161	0.7885	0.107*	
C13	−0.1357 (5)	−0.2210 (10)	0.8991 (5)	0.0940 (15)	
H13	−0.1374	−0.3023	0.9615	0.113*	
C14	−0.1515 (5)	−0.0390 (8)	0.9098 (5)	0.0903 (16)	
H14	−0.1651	0.0056	0.9794	0.108*	
C15	−0.1479 (4)	0.0835 (6)	0.8193 (4)	0.0702 (11)	
H15	−0.1579	0.2101	0.8289	0.084*	
H1A	0.102 (3)	0.001 (3)	0.614 (2)	0.049 (7)*	

### Atomic displacement parameters (Å^2^)

**Table:**

	*U*^11^	*U*^22^	*U*^33^	*U*^12^	*U*^13^	*U*^23^
S1	0.0470 (4)	0.0432 (4)	0.0420 (3)	0.0052 (4)	0.0189 (3)	0.0057 (4)
O1	0.0850 (19)	0.095 (2)	0.0564 (14)	−0.0256 (17)	0.0441 (13)	−0.0240 (15)
O2	0.0750 (16)	0.0817 (16)	0.0677 (13)	−0.0294 (18)	0.0455 (12)	−0.0137 (18)
O3	0.0640 (16)	0.0639 (16)	0.0641 (15)	0.0019 (13)	0.0206 (12)	0.0255 (13)
N1	0.0404 (14)	0.0454 (15)	0.0379 (12)	−0.0008 (11)	0.0146 (11)	−0.0041 (11)
C1	0.0383 (15)	0.0425 (17)	0.0387 (14)	−0.0046 (13)	0.0168 (12)	−0.0055 (13)
C2	0.0424 (17)	0.060 (2)	0.0489 (18)	−0.0037 (16)	0.0205 (14)	−0.0013 (16)
C3	0.098 (4)	0.119 (5)	0.086 (3)	−0.040 (3)	0.065 (3)	−0.019 (3)
C4	0.105 (4)	0.132 (5)	0.112 (4)	−0.035 (4)	0.070 (3)	0.001 (3)
C5	0.0499 (18)	0.0452 (17)	0.0415 (16)	0.0018 (15)	0.0132 (13)	−0.0001 (14)
C6	0.048 (2)	0.073 (2)	0.064 (2)	0.0088 (19)	0.0065 (17)	0.002 (2)
C7	0.067 (2)	0.048 (3)	0.067 (2)	0.0015 (18)	0.0159 (17)	−0.0079 (17)
C8	0.080 (3)	0.067 (2)	0.0490 (19)	0.004 (2)	0.0276 (18)	0.0121 (18)
C9	0.0421 (17)	0.065 (2)	0.0451 (16)	0.0087 (16)	0.0174 (14)	0.0066 (15)
C10	0.0369 (17)	0.059 (2)	0.0522 (18)	−0.0030 (14)	0.0190 (14)	0.0005 (15)
C11	0.062 (2)	0.070 (3)	0.064 (2)	−0.0145 (19)	0.0243 (18)	−0.0035 (18)
C12	0.092 (4)	0.058 (3)	0.113 (4)	−0.018 (2)	0.038 (3)	0.011 (2)
C13	0.086 (3)	0.108 (4)	0.094 (3)	−0.021 (4)	0.044 (2)	0.033 (4)
C14	0.095 (4)	0.121 (5)	0.077 (3)	−0.006 (3)	0.057 (3)	0.012 (3)
C15	0.074 (3)	0.075 (3)	0.074 (2)	−0.001 (2)	0.043 (2)	−0.002 (2)

### Geometric parameters (Å, °)

**Table:**

S1—O3	1.487 (2)	C6—H6C	0.9600
S1—N1	1.639 (3)	C7—H7A	0.9600
S1—C9	1.824 (4)	C7—H7B	0.9600
O1—C2	1.201 (4)	C7—H7C	0.9600
O2—C2	1.316 (4)	C8—H8A	0.9600
O2—C3	1.471 (4)	C8—H8B	0.9600
N1—C1	1.455 (4)	C8—H8C	0.9600
N1—H1A	0.859 (17)	C9—C10	1.480 (5)
C1—C2	1.523 (4)	C9—H9A	0.9700
C1—C5	1.555 (4)	C9—H9B	0.9700
C1—H1	0.9800	C10—C11	1.371 (5)
C3—C4	1.439 (7)	C10—C15	1.398 (5)
C3—H3A	0.9700	C11—C12	1.382 (6)
C3—H3B	0.9700	C11—H11	0.9300
C4—H4A	0.9600	C12—C13	1.375 (7)
C4—H4B	0.9600	C12—H12	0.9300
C4—H4C	0.9600	C13—C14	1.334 (8)
C5—C7	1.520 (5)	C13—H13	0.9300
C5—C8	1.524 (5)	C14—C15	1.374 (6)
C5—C6	1.526 (5)	C14—H14	0.9300
C6—H6A	0.9600	C15—H15	0.9300
C6—H6B	0.9600		
			
O3—S1—N1	112.37 (15)	H6A—C6—H6C	109.5
O3—S1—C9	104.90 (15)	H6B—C6—H6C	109.5
N1—S1—C9	96.19 (15)	C5—C7—H7A	109.5
C2—O2—C3	116.2 (3)	C5—C7—H7B	109.5
C1—N1—S1	117.2 (2)	H7A—C7—H7B	109.5
C1—N1—H1A	111.1 (19)	C5—C7—H7C	109.5
S1—N1—H1A	120.2 (19)	H7A—C7—H7C	109.5
N1—C1—C2	110.4 (2)	H7B—C7—H7C	109.5
N1—C1—C5	111.9 (2)	C5—C8—H8A	109.5
C2—C1—C5	112.4 (3)	C5—C8—H8B	109.5
N1—C1—H1	107.3	H8A—C8—H8B	109.5
C2—C1—H1	107.3	C5—C8—H8C	109.5
C5—C1—H1	107.3	H8A—C8—H8C	109.5
O1—C2—O2	123.9 (3)	H8B—C8—H8C	109.5
O1—C2—C1	124.3 (3)	C10—C9—S1	112.7 (2)
O2—C2—C1	111.8 (3)	C10—C9—H9A	109.1
C4—C3—O2	107.8 (4)	S1—C9—H9A	109.1
C4—C3—H3A	110.1	C10—C9—H9B	109.1
O2—C3—H3A	110.1	S1—C9—H9B	109.1
C4—C3—H3B	110.1	H9A—C9—H9B	107.8
O2—C3—H3B	110.1	C11—C10—C15	117.5 (4)
H3A—C3—H3B	108.5	C11—C10—C9	121.1 (3)
C3—C4—H4A	109.5	C15—C10—C9	121.4 (3)
C3—C4—H4B	109.5	C10—C11—C12	121.0 (4)
H4A—C4—H4B	109.5	C10—C11—H11	119.5
C3—C4—H4C	109.5	C12—C11—H11	119.5
H4A—C4—H4C	109.5	C13—C12—C11	119.8 (4)
H4B—C4—H4C	109.5	C13—C12—H12	120.1
C7—C5—C8	109.2 (3)	C11—C12—H12	120.1
C7—C5—C6	109.4 (3)	C14—C13—C12	120.2 (4)
C8—C5—C6	110.6 (3)	C14—C13—H13	119.9
C7—C5—C1	111.6 (2)	C12—C13—H13	119.9
C8—C5—C1	107.2 (3)	C13—C14—C15	120.8 (5)
C6—C5—C1	108.8 (3)	C13—C14—H14	119.6
C5—C6—H6A	109.5	C15—C14—H14	119.6
C5—C6—H6B	109.5	C14—C15—C10	120.8 (4)
H6A—C6—H6B	109.5	C14—C15—H15	119.6
C5—C6—H6C	109.5	C10—C15—H15	119.6
			
O3—S1—N1—C1	84.8 (2)	N1—C1—C5—C6	172.6 (3)
C9—S1—N1—C1	−166.3 (2)	C2—C1—C5—C6	47.8 (3)
S1—N1—C1—C2	−95.2 (3)	O3—S1—C9—C10	−179.6 (3)
S1—N1—C1—C5	138.8 (2)	N1—S1—C9—C10	65.2 (3)
C3—O2—C2—O1	−2.6 (6)	S1—C9—C10—C11	−99.0 (4)
C3—O2—C2—C1	178.3 (4)	S1—C9—C10—C15	78.2 (4)
N1—C1—C2—O1	−51.2 (4)	C15—C10—C11—C12	0.1 (6)
C5—C1—C2—O1	74.6 (4)	C9—C10—C11—C12	177.4 (4)
N1—C1—C2—O2	128.0 (3)	C10—C11—C12—C13	0.1 (7)
C5—C1—C2—O2	−106.3 (3)	C11—C12—C13—C14	0.2 (8)
C2—O2—C3—C4	178.3 (4)	C12—C13—C14—C15	−0.7 (9)
N1—C1—C5—C7	51.8 (3)	C13—C14—C15—C10	0.9 (7)
C2—C1—C5—C7	−73.1 (4)	C11—C10—C15—C14	−0.5 (6)
N1—C1—C5—C8	−67.8 (3)	C9—C10—C15—C14	−177.8 (4)
C2—C1—C5—C8	167.4 (3)		

### Hydrogen-bond geometry (Å, °)

**Table:**

*D*—H···*A*	*D*—H	H···*A*	*D*···*A*	*D*—H···*A*
C7—H7B···O1	0.96	2.61	3.234 (5)	123.
C9—H9B···O3^i^	0.97	2.48	3.296 (5)	142.
N1—H1A···O3^i^	0.86 (2)	2.13 (2)	2.932 (3)	156 (3)

Symmetry codes: (i) −*x*, *y*−1/2, −*z*+1.
